# Finafloxacin, a Novel Fluoroquinolone, Reduces the Clinical Signs of Infection and Pathology in a Mouse Model of Q Fever

**DOI:** 10.3389/fmicb.2021.760698

**Published:** 2021-11-30

**Authors:** M. Gill Hartley, Isobel H. Norville, Mark I. Richards, Kay B. Barnes, Kevin R. Bewley, Julia Vipond, Emma Rayner, Andreas Vente, Stuart J. Armstrong, Sarah V. Harding

**Affiliations:** ^1^CBR Division, Defence Science and Technology Laboratory (Dstl), Porton Down, Salisbury, United Kingdom; ^2^College of Life and Environmental Sciences, University of Exeter, Exeter, United Kingdom; ^3^Public Health England, Porton Down, Salisbury, United Kingdom; ^4^MerLion Pharmaceuticals, Berlin, Germany

**Keywords:** *Coxiella*, antibiotics, bacterial counts, splenomegaly, PCR

## Abstract

Finafloxacin is a novel fluoroquinolone with optimal antibacterial activity in low pH environments, therefore offering a therapeutic advantage over some traditional antibiotics, in treating bacterial infections associated with acidic foci. *Coxiella burnetii*, the causative agent of Q fever, is a bacterium which resides and replicates in acidic intracellular parasitic vacuoles. The efficacy of finafloxacin was evaluated *in vivo* using the A/J mouse model of inhalational Q fever and was compared to doxycycline, the standard treatment for this infection and ciprofloxacin, a comparator fluoroquinolone. Finafloxacin reduced the severity of the clinical signs of infection and weight loss associated with Q fever, but did not reduce the level of bacterial colonization in tissues compared to doxycycline or ciprofloxacin. However, histopathological analysis suggested that treatment with finafloxacin reduced tissue damage associated with *C. burnetii* infection. In addition, we report for the first time, the use of viable counts on axenic media to evaluate antibiotic efficacy *in vivo*.

## Introduction

Q fever is a zoonotic disease, presenting in 40% of human cases as an acute pulmonary infection characterized by a self-limiting febrile illness (Smith et al., 1993). The primary reservoir is ruminants, such as goats and cows, concentrating in birth products and infecting humans mainly via aerosol transmission. The largest recorded outbreak of Q fever occurred in the Netherlands, was associated with infected dairy farms, and caused overt illness in over 4,000 people between 2007 and 2011 (Roest et al., 2011). The causative agent, *Coxiella burnetii* is an intracellular Gram-negative bacterium that is difficult to culture (Omsland et al., 2011), consequently the disease is difficult to diagnose, frequently presenting with non-specific symptoms. Confirmation of diagnoses currently relies upon a combination of PCR and serology (Fournier et al., 1998; Turra et al., 2006; Anderson et al., 2013). Although some cases may resolve without treatment, for others a delay in treatment or poor/inappropriate treatment of acute disease can lead to long-term life altering complications, such as chronic fatigue syndrome and endocarditis (van Loenhout et al., 2012).

Doxycycline, is the standard treatment for acute Q fever and has been shown to reduce the duration of fever (Anderson et al., 2013). However, doxycycline is often poorly tolerated with adverse reactions reported by up to 66% of patients, which include abdominal pain, nausea and vomiting (Järvinen et al., 1995; Maurin and Raoult, 1999; Saunders et al., 2015). Failure to complete the 2-week course of doxycycline can result in “relapse” of disease (Benenson and Tigertt, 1956). Chronic Q fever can manifest years after the acute infection (Maurin and Raoult, 1999) and is twice as likely to occur from undiagnosed, untreated disease (Kampschreur et al., 2014). It can be a life-threatening condition, especially when combined with other co-morbidities, such as vascular disease (van Roeden et al., 2019). Chronic Q fever is currently treated with a doxycycline/hydroxychloroquine combination for extended periods, often in excess of 18 months (Maurin and Raoult, 1999). The need for alternative treatment options for Q fever is further heightened by the isolation of a strain demonstrating doxycycline resistance (Rolain et al., 2005).

*C. burnetii* is an intracellular pathogen, residing and replicating in acidic parasitic vacuoles (Voth and Heinzen, 2007). Typically, the pH within the vacuoles is less than 5 which can reduce antibiotic activity. It is suggested that the benefit of the hydroxychloroquine/doxycycline combination therapy is as a result of the ability of hydroxychloroquine to raise the pH of the parasitic vacuoles to a point where doxycycline is active (Maurin et al., 1992; Omsland et al., 2009; Smith et al., 2019).

Finafloxacin is a novel fluoroquinolone that has recently undergone Phase II clinical trials for the treatment of urinary tract infections in hospitalized patients (Wagenlehner et al., 2018). It has enhanced *in vitro* activity in acidic conditions (Smith et al., 1988; Stubbings et al., 2011) and is efficacious against a number of Gram negative and Gram positive organisms including *Acinetobacter baumannii, Staphylococcus aureus, Burkholderia pseudomallei*, and *Francisella tularensis* (Higgins et al., 2010; Lemaire et al., 2011; Barnes et al., 2017, 2019a,b). Finafloxacin has demonstrated good cellular penetration and at acidic pH, an increased accumulation within eukaryotic cells (Lemaire et al., 2011; Chalhoub et al., 2020). As *C. burnetii* resides solely within acidic parasitic vacuoles, the use of finafloxacin may have advantages over antibiotics such as doxycycline.

Since the successful formulation of axenic media with the ability to support the growth of *C. burnetii*, direct *in vitro* evaluation of antibiotics against the bacteria has been simplified (Omsland et al., 2009; Kersh, 2013; Clay et al., 2018; Smith et al., 2019) but few *in vivo* antibiotic efficacy studies have been undertaken to explore alternative treatment options. This work evaluates the efficacy of finafloxacin *in vivo* using the A/J mouse model of inhalational Q fever and assesses the outcome of treatment using multiple parameters including clinical signs of infection; organ weight; bacterial colonization (by RT-PCR and viable count); and the histopathological analyses of organs.

## Materials and Methods

### Bacteria

*C. burnetii* strain Nine Mile Phase I (RSA 493) was grown axenically in 100 mL acidified citrate cysteine medium-2 (ACCM-2), in 250 mL Erlenmeyer flasks (Omsland et al., 2009). Bacterial cultures were incubated at 37°C, shaking at 75 rpm for 6 days, in a biojar containing a GENbox microaer generator (bioMérieux, France) to displace oxygen. Following incubation, bacteria were harvested by centrifugation at 10 000 × *g* at 21°C for 20 min and re-suspended in sterile phosphate-buffered saline (PBS) at a concentration of approximately 1 × 10^9^ genome equivalents (GE)/mL.

Viable bacteria were enumerated by serial dilution in PBS, plated onto ACCM-2 agarose plates (Sunrise Science Products, San Diego) and incubated as previously described for 14 days (Hartley et al., 2019).

### Bacterial Enumeration Using Real Time Quantitative PCR

*C. burnetii* was enumerated using quantitative RT- PCR targeting the *icd* gene using the forward primer: GTTCCCAGCC AAGGTGAAAA and the reverse primer: GGGTCGGTCAG GAACTTCTAAA. The sequence of the probe was ATCACCGTTAATAAAGC, covalently labeled at the 5′ end with the reporter dye FAM and at the 3′ end with the quencher dye BHQ-1™ (Sigma, United Kingdom). Bacterial chromosomal DNA was extracted using QIAGen QIAmp DNA Mini kit/Blood Mini/Tissue (Qiagen) depending on sample type. A typical *real time quantitative* PCR (RT-PCR) reaction comprised of 2 μL template DNA, forward primer (900 nM), reverse primer (900 nM), probe (250 nM) and a Fast TaqMan mastermix (Thermofisher). PCR cycling conditions were as follows: 3 min at 95°C, 30 s at 60°C, followed by 50 two-step cycles of 15 s at 95°C and 30 s at 60°C. For each PCR reaction, a control of linearized synthetic plasmid containing a single copy of the target was included. This was quantitated after linearization and purification using a ND-2500 NanoDrop spectrophotometer. For each reaction, a standard curve of the control plasmid was run in duplicate in the range 1 × 10^7^–1 × 10^2^ GE/mL. A plasmid concentration of 1 × 10^1^ GE/mL was included (in triplicate) as a lower-limit check in each assay.

### Antibiotics

Finafloxacin was supplied by MerLion Pharmaceuticals Pte Ltd. A 15 mg/mL solution was prepared by adding 2.1 mL of 0.01 M Tris buffer to 44 mg of finafloxacin powder (containing 37.5 mg of active ingredient). Two hundred microliters of 1 M sodium hydroxide was added to dissolve the antibiotic followed by 200 μL of 0.015 M hydrochloric acid, to give a final pH of 8. An intravenous preparation of ciprofloxacin (Ciproxin^®^ 2 mg/mL) was purchased from Bayer (Basingstoke, United Kingdom). Doxycycline hyclate (Sigma Aldrich, United Kingdom) was dissolved in distilled water to a working concentration of 42 mg/mL.

### Determination of the Minimum Inhibitory Concentration

The lowest concentration of finafloxacin that prevented visible growth of *C. burnetii* in ACCM-2 was determined as per Clay et al. (2018), following the Clinical and Laboratory Standard Institute guidelines. Briefly, broths containing finafloxacin (1 μg/mL–1 ng/mL) were inoculated with approximately 5 × 10^6^/mL (final concentration) of *C. burnetii* and incubated for 6 days at 37 °C in 5% CO_2_/2.5% O_2_. Optical density (600 nm) was measured at 6 days following inoculation. The assay was performed in triplicate.

### Animal Studies

#### Animals

The animal studies were carried out in accordance with the United Kingdom Animal (Scientific Procedures) Act (1986) and the Codes of Practice for the Housing and Care of Animals used in Scientific Procedures, 1989. Male A/J mice (Envigo, Huntington Life sciences, United Kingdom) weighing 16–20 g were randomized into cages of 5 within an ACDP (United Kingdom) Level 3 flexible-film isolator in an ACDP Level 3 laboratory and were housed on a 12 h day-night light cycle, with food and water available *ad libitum*. Mice were allowed to acclimatize for 1 week before any procedures were performed.

#### Pharmacokinetics of Finafloxacin

Finafloxacin was administered to 30 mice by the oral route at a dose of 37.5 mg/kg. Blood was collected via cardiac puncture under terminal anesthesia into lithium heparin tubes from groups of three mice at 15, 30 min, 1, 1.5, 2, 3, 4, 6, 8, 24 h post-dosing. The samples were centrifuged to separate the plasma from the whole blood and stored at −80°C until analysis, by high-performance liquid chromatography (HPLC). Separation was achieved by using reverse phase chromatography with a gradient of acetonitrile/methanol (75/25, v/v) containing 0.1 % formic acid. Detection was achieved with triple-stage quadrupole MS/MS in the selected reaction monitoring mode (Swiss BioQuant AG, Switzerland). Pharmacokinetic analysis of the mean concentration-time profile data was completed using Phoenix WinNonlin (Phoenix, v 6.1 Pharsight Corp., United States), this included non-compartmental and compartmental analysis. The pharmacokinetic parameters; apparent volume of distribution (V), terminal half-life (t½), rate of clearance (CL), maximum concentration (C_max_), time of maximum concentration (T_max_) and area under the concentration-time curve (AUC) were determined by non-compartmental pharmacokinetic analysis using WinNonlin Phoenix (v 6.1 Pharsight Corp., United States). The doxycycline and ciprofloxacin doses and treatment regimens used were taken from Norville et al. (2014).

The clearance rate of finafloxacin in the mice was subsequently used to calculate a murine human-equivalent dose (Equation 1). A concentration-time profile in mice was simulated for the human-equivalent dose (30 mg/kg, once daily) which was then simulated by using a compartmental model (Phoenix WinNonLin) to verify the AUC.


(1) $$Dose=AUC_{target}\times Clearance_{murine}$$

The pharmacokinetic guided approach to dose extrapolation, adapted from Sharma and McNeill (2009).

#### Infection of Mice and Antibiotic Efficacy Studies

Mice were challenged with an aerosol generated using the AeroMP-Henderson apparatus, and a six-jet Collison nebulizer (BGI, Waltham, MA) operating at 15 L/min, 65% humidity, room temperature. The aerosol was mixed with conditioned air in the spray tube and delivered via a head-only exposure chamber. Samples of the aerosol were taken using an AGI-30 (Ace Glass Inc., United States) containing PBS at 6 L/min and an aerodynamic particle sizer (TSI Instruments, Ltd., Bucks, United Kingdom); these processes were controlled and monitored using the AeroMP management platform (Biaera Technologies, LLC, Frederick, MD). A back titration of the bacterial culture taken at the time of challenge was performed using RT-PCR as described above. Direct bacterial enumeration was used to calculate the presented dose using a derived respiratory minute volume of 19.9 mL estimated using the average weight of the animals (Guyton, 1947).

#### Determination of the Efficacy of 7 Days of Treatment With Finafloxacin, Ciprofloxacin or Doxycycline

Groups of ten infected mice were treated from 24 h post-challenge (p.c.) for 7 days with 40 μL of finafloxacin (30 mg/kg) administered orally once a day, 220 μL of ciprofloxacin (22 mg/kg) administered by the intraperitoneal route twice daily (12 h intervals), or 50 μL of doxycycline hyclate (105 mg/kg) administered orally twice daily (12 h intervals). Control groups of infected mice were dosed with 40 μL of diluent (consisting of Tris buffer, sodium hydroxide and hydrochloric acid), described as the finafloxacin carrier, or 40 μL of sterile PBS orally once a day. One group of uninfected mice were administered 40 μL of finafloxacin orally to evaluate tolerability.

Mice were observed twice daily for clinical signs of disease (piloerection, arched back, dehydration, eye problems, wasp-waisted, immobility) and weighed every morning for 14 days. At day 14 p.c. all mice were euthanized and the lungs and spleens aseptically removed and weighed. Half the organ was placed into 10% neutral buffered formalin for histopathological analysis and half was stored at −80^°^C until required. Samples were thawed and homogenized using a tissue homogenizer (Precellys^®^ 24, Bertin instruments) into 1 mL sterile PBS. The colonizing bacteria were enumerated using RT-PCR or by plating 100 μL volumes onto ACCM-2 plates as described above.

#### Pathogenesis Study of the Efficacy of 7 Days of Treatment With Finafloxacin, With Extended Monitoring for 28 Days Post-challenge

Groups of 10 infected mice were treated from 24 h p.c. for 7 days with 40 μL of finafloxacin (30 mg/kg) or 40 μL of carrier administered orally once a day. Mice were monitored as above. At days 4, 8, 14, 21, and 28 p.c., mice were euthanized and lungs and spleens aseptically removed. Tissues were processed for bacteriology and histopathological analysis as described above.

#### Efficacy of 7 vs. 14 Days of Treatment With Finafloxacin, With Extended Monitoring for 35 Days Post-challenge

Groups of 10 mice were infected and treated from 24 h p.c. for 7 or 14 days with 40 μL of finafloxacin (30 mg/kg) or 40 μL of the carrier administered orally once a day. Mice were monitored as in study 1. Mice treated for 7 days were euthanized at day 8 and 28 p.c. and mice treated for 14 days at day 15, 28, and 35 p.c., and the lungs and spleens aseptically removed. Tissues were processed for bacteriology and histopathological analysis as described above.

### Histopathology

Samples of the spleens and lungs (in the later studies) were processed and embedded in paraffin wax. Sections were cut at 5–6 μm and stained with hematoxylin and eosin. The appearance of the tissues were recorded as: within normal limits (where *C. burnetii-*associated lesions were not detected) or positive (where *C. burnetii-*associated lesions were detected). Slides were assessed independently by two pathologists who were blinded to the study details.

### Statistical Analysis

All statistical analysis and graphs were produced using Prism (v8 GraphPad Software Inc.). Data analysis (comparison of body weight change, organ weight change and bacterial loads) compared to the control animals, was carried out using one way ANOVA or ANOVA mixed effects model analysis, after conformation of normal distribution of the data by data residual analysis. Where only two groups were considered, significance was tested using a student *t*-test with Bonferroni corrections. For the presence or absence of histology lesions, a multiple Fisher exact test with Bonferroni correction was used. Significance markers were: *p < 0.05; ^**^p < 0.01; ^***^p < 0.001.

## Results

### Minimum Inhibitory Concentration

The Minimum inhibitory concentration (MIC) of finafloxacin for *C. burnetii* Nine Mile Phase I (RSA 493), after 6 days growth in ACCM-2 broth, was determined as 0.03 μg/mL, at a pH of less than 5.

### Pharmacokinetics of Finafloxacin

The concentration-time profile of finafloxacin in male A/J mice (*n* = 30) and different parameters were determined by non-compartmental analysis (Table 1).

**TABLE 1:** Non-compartmental parameter estimates for finafloxacin in male A/J mice, following administration of finafloxacin by the oral route (37.5 mg/kg).

Parameter	Value
V	2.65 L/kg
t½	1.86 h
CL	0.99 L/h/kg
AUC	37.10 mg⋅h/L
C_max_	7.6 mg/L
T_max_	1.5 h

*Analysis was completed using Phoenix WinNonlin (v6.1 Certara Inc.). V, Apparent volume of distribution; t½, terminal half-life; CL, rate of clearance; AUC, area under the concentration-time curve; C_max_, maximum concentration; T_max_, time of maximum concentration.*

A human-equivalent finafloxacin dose for use in the efficacy studies was calculated as the product of finafloxacin clearance and the human AUC (29.2 ± 7.8 mg⋅h/L, AUC_0–∞_ following a single 800 mg dose of finafloxacin; Patel et al., 2011) using Equation 1(*Dose* = *AUC*_*target*_×*Clearance*_*murine*_). Simulation of this human-equivalent dose (30 mg/kg once a day) with a one-compartment model predicted the equivalent human exposure (33.6 mg⋅h/L) would be achieved in mice.

### Determination of the Efficacy of 7 Days of Treatment With Finafloxacin, Ciprofloxacin or Doxycycline

**FIGURE 1:**
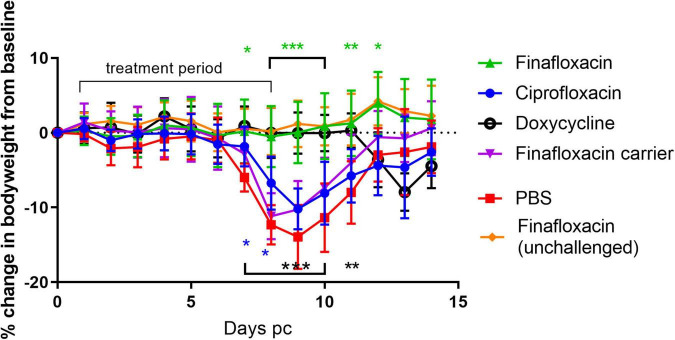
Bodyweight loss in mice infected with *C. burnetii* and treated with antibiotics. At 24 h post-challenge mice were treated with finafloxacin (30 mg/kg once a day orally), ciprofloxacin (22 mg/kg every 12 h via intraperitoneal injection) or doxycycline (105 mg/kg once daily orally) for 7 days. Control animals received PBS or the carrier orally. One group of mice received finafloxacin only (not challenged). Mean and standard errors are shown. Statistical difference from PBS treated controls, *p < 0.05, **p < 0.01***p < 0.001, color denotes group.

To determine the efficacy of finafloxacin *in vivo*, mice were infected by the aerosol route with a presented (inhaled) dose of *C. burnetii* at a concentration of 1.6 × 10^6^ GE/mL. and treated at 24 h p.c with finafloxacin, ciprofloxacin or doxycycline for 7 days. As the key clinical parameter in this model is weight loss (Norville et al., 2014), animals were weighed once daily (Figure 1). Mice that were infected with *C. burnetii* and received PBS or the finafloxacin carrier lost 15 or 12% of their body weight, respectively, by day 9 p.c. In addition, 90% of animals exhibited clinical signs of disease including piloerection and arched backs. Mice that received ciprofloxacin initially had less weight loss (day 7 and 8, *p* < 0.05) but thereafter their weight loss was comparable to the PBS group, with a 10% loss observed by day 9 p.c., and with 80% showing clinical signs. In comparison, mice receiving finafloxacin or doxycycline were protected from a loss of bodyweight significant from day 8 p.c. (*p* < 0.001) and had reduced clinical signs. On day 11 p.c., 3 days after the cessation of treatment, the doxycycline treated mice started to lose weight, peaking at 8% on day 13 p.c., with no further clinical signs reported. No weight loss was observed for the mice treated with finafloxacin.

**FIGURE 2:**
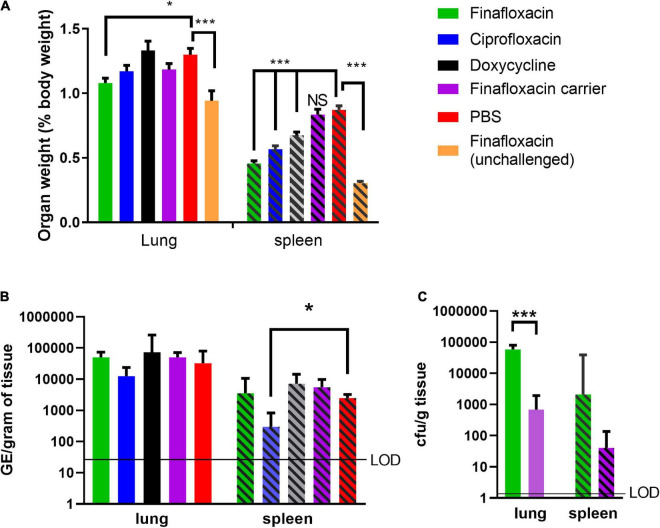
Organ weight and bacterial load at day 14 post-challenge in mice challenged with *C. burnetii* and treated with antibiotics. At 24 h post-challenge mice were treated with finafloxacin (30 mg/kg once a day orally), ciprofloxacin (22 mg/kg every 12 h via intraperitoneal injection) or doxycycline (105 mg/kg once daily orally) for 7 days. Control animals received PBS or the carrier orally. One group of mice received finafloxacin and were unchallenged. **(A)** Lung and spleen weight as a percentage of total body weight, **(B)** bacterial load in lungs and spleens assessed by RT PCR, and **(C)** bacterial load in the lungs and spleens from animals treated with finafloxacin or carrier determined by viable count. Geometric mean and SDs are shown, limit of detection marked LOD. *p < 0.05, ***p < 0.001.

Another key indicator of Q fever disease is enlarged organs, specifically splenomegaly and, following aerosol infection, also the lung. At day 14 p.c. (when the study was terminated), the lungs and spleens from animals that received PBS or the carrier were enlarged, compared to those from the unchallenged finafloxacin treated animals (*p* < 0.001; Figure 2A). Treatment with finafloxacin, ciprofloxacin, or doxycycline, significantly moderated splenomegaly (*p* < 0.001), when compared to those from animals treated with PBS. The lungs from animals treated with finafloxacin were also smaller than those from the PBS treated group (*p* = 0.033).

The bacterial load in the lungs and spleens were determined by RT-PCR, which has historically been used to enumerate *C. burnetii* genomic equivalents (GEs) by measuring total bacterial DNA (Norville et al., 2014). Only mice treated with ciprofloxacin had a lower concentration of bacteria in the spleen than those treated with PBS (*p* = 0.035; Figure 2B). No differences in the bacterial load of the lungs or the spleens were observed between mice treated with finafloxacin or doxycycline and those treated with PBS or the finafloxacin carrier. Viable counts using axenic media were also performed on tissue samples from the finafloxacin and carrier treated groups, which had higher bacterial loads in both the spleen and lungs from the mice treated with finafloxacin (lung *p* < 0.001; Figure 2C).

Histopathological analyses was performed on sections of the spleens from all animals. All groups showed some degree of splenic tissue damage typical of *C. burnetii* colonization. There was a trend observed that suggested that these lesions were fewer and less severe in spleens harvested from the finafloxacin or ciprofloxacin treated groups (data not shown).

### Pathogenesis Study of the Efficacy of 7 Days Treatment With Finafloxacin, With Extended Monitoring for 28 Days Post-challenge

To further understand the efficacy provided by finafloxacin, mice were challenged with a presented (inhaled) dose of *C. burnetii* at 9.4 × 10^6^ GE/mL. and treated with finafloxacin or the carrier for 7 days. Clinical signs and weight loss were recorded daily. On days 4, 8, 14, 21, and 28 p.c., groups of mice were euthanized, the lungs and spleens aseptically removed and the bacterial load determined using RT-PCR. Bacterial colonization was also determined by viable count on day 14 p.c.

**FIGURE 3:**
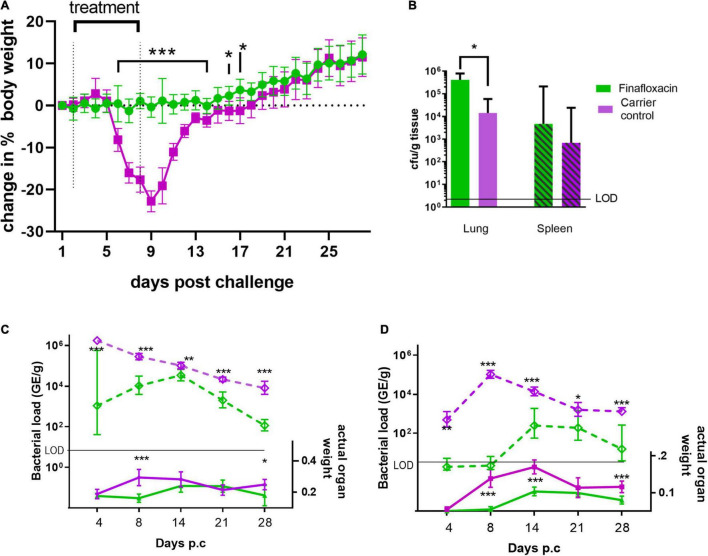
Body weight, organ weight and bacterial burden in mice infected with *C. burnetii* and treated with finafloxacin for 7 days. At 24 h post-challenge mice were treated with finafloxacin (30 mg/kg) or the carrier once a day orally for 7 days. **(A)** Body weight change from baseline, **(B)** bacterial burden for lung and spleen determined by viable counts on day 14 p.c. **(C)** Actual Lung weight (solid line) and bacterial burden assessed by RT-PCR (dotted line) on days 4, 8, 14, 21 and 28 p.c. **(D)** Actual spleen weight (solid line) and bacterial burden assessed by RT-PCR (dotted line) on days 4, 8, 14, 21 and 28 pc. Mean and SEs are shown for weights, geometric mean and SD for GE or cfu, limit of detection marked LOD. *p < 0.05, **p < 0.01, ***p < 0.001.

As in the first study, mice infected with *C. burnetii* and treated with the carrier lost weight, the peak observed at day 9, with 100% of the animals displaying clinical signs of disease. Infected mice treated with finafloxacin did not lose weight (significantly different between day 6 and 14 p.c, *p* < 0.001 and day 16 and 17 *p* < 0.05 Figure 3A). Transient piloerection was observed in 10% of these treated mice.

Both the lungs and spleens from animals treated with the carrier were significantly enlarged compared to those treated with finafloxacin at day 8 p.c. (*p* < 0.001) but not at day 4. This was true, when the organ weight was assessed as either percentage of body weight or by actual organ weight (Figures 3B,C). After day 14 the size of the carrier treated organs declined although had not returned to normal by day 28 p.c Interestingly, enlargement of these organs was also observed from the animals treated with finafloxacin following cessation of treatment, with significant splenomegaly on days 14 and 21 p.c. (when compared to day 4, *p* < 0.001). This correlates with the bacterial burden determined by RT-PCR in the organs of the treated mice, which was highest at day 14 p.c. (Figures 3C,D). However, the disease in those animals treated with finafloxacin was not as severe as that observed in the animals treated with the carrier (when organ weights, bacterial load and clinical signs were compared).

To evaluate bacterial clearance, sections of the lungs and spleens collected on day 14 p.c. were also cultured to obtain viable counts, which showed that both the lungs and spleens from the finafloxacin treated animals were more heavily colonized than those from the carrier treated animals (significant for the lungs *p* = 0.012; Figure 3B).

**FIGURE 4:**
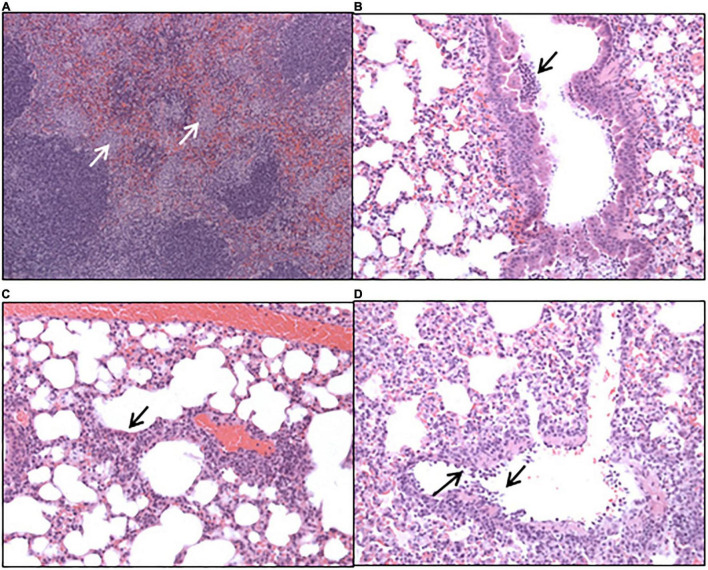
Hematoxylin-and-eosin-stained tissues harvested from mice infected with *C. burnetii* and treated with the vehicle control. **(A)** Spleen harvested at day 8 post-challenge showing marked, multifocal, granulomatous splenitis **(B)** lung harvested at day 4 post-challenge showing acute, focal bronchiolitis and **(C)** acute, focal alveolitis. **(D)** Lung harvested at day 8 post-challenge showing vasculitis.

**FIGURE 5:**
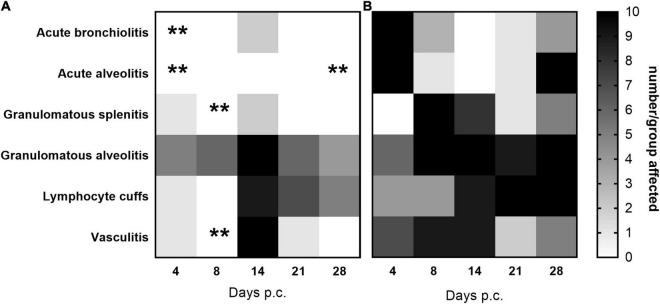
The occurrence of typical histopathological features associated with *C. burnetii* in lung and spleen tissues. At 24 h post-challenge mice were treated with **(A)** finafloxacin (30 mg/kg) or **(B)** the carrier, orally daily for 7 days. Tissues were taken on days 4, 8, 14, 21, and 28 p.c. and scored for the presence/absence of 6 typical histopathological features associated with murine Q fever, and the occurrence displayed as a heat map (where white is no animal affected and black is all 10 affected). **p < 0.01.

Histopathological analysis was performed on sections of tissues taken at all-time points during the study. Each tissue was examined for the presence of 6 typical features associated with murine Q fever (vasculitis, lymphocyte cuffs, acute bronchiolitis, acute alveolitis, and granulomatous alveolitis (in the lungs), or granulomatous splenitis (in the spleen), representative images of some of these features are shown in Figure 4). The occurrence of these features is illustrated in Figure 5. All animals treated with the carrier had some evidence of *C. burnetii-*associated lesions at each time point. In comparison, tissues from animals treated with finafloxacin had reduced damage at all-time points except on day 14 p.c., when all animals had evidence of granulomatous alveolitis and vasculitis, which correlated with the peak in organ weight and the highest bacterial burden. Treatment with finafloxacin did confer some protection against splenic damage (significantly different on day 8 p.c. *p* < 0.01). It was also apparent that the tissue damage from animals treated with finafloxacin was minor and transient in nature, by day 28 p.c., 50% of these animals did not show any evidence of tissue damage.

### Efficacy of 7 vs. 14 Days Treatment With Finafloxacin, With Extended Monitoring to 35 Days Post-challenge

**FIGURE 6:**
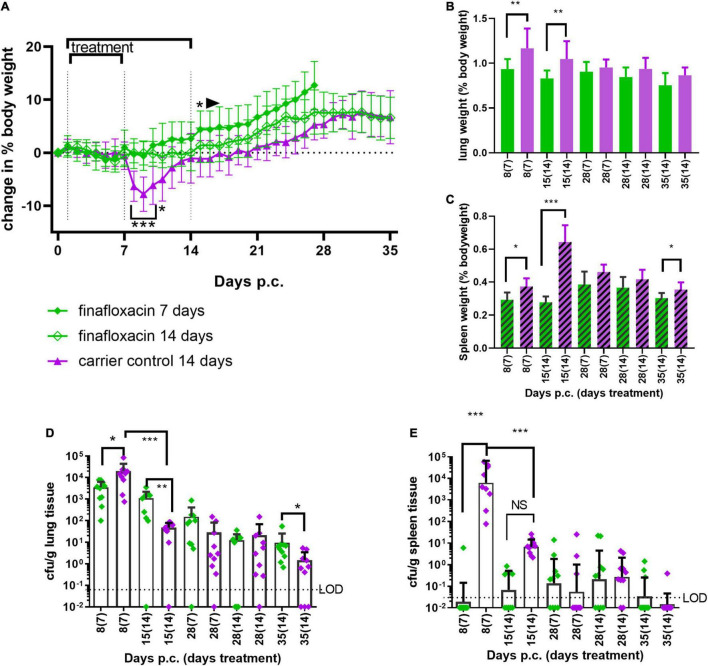
Efficacy of 7 or 14 days of finafloxacin treatment following infection with *C. burnetii*. At 24 h post-challenge mice were treated with finafloxacin (30 mg/kg) or the carrier, once a day orally for 7 or 14 days. **(A)** Weight loss compared to baseline, **(B)** lung weight as a percentage of body weight, **(C)** spleen weight as a percentage of body weight, **(D)** bacterial burden for lung by viable count, and **(E)** bacterial burden for spleen by viable count. Mean and SEs are shown for weights, geometric mean and SD for cfu, limit of detection marked LOD. *p < 0.05, **p < 0.01, ***p < 0.001.

Doxycycline is normally prescribed for 2 weeks to treat human Q fever. Therefore, to determine if bacterial clearance could be improved by a longer treatment regimen, mice were treated with finafloxacin or the carrier for 7 or 14 days. Groups of mice were challenged with a presented (inhaled) dose of *C. burnetii* at a concentration of 1 1.5 × 10^6^ GE/mL and euthanized immediately following cessation of treatment (day 8 and 15 p.c.), at day 28 and 35 days p.c. Lungs and spleens were harvested, weighed and the bacterial burden determined by viable count. Mice treated with the carrier lost 10% of their body weight, peaking at day 8 p.c, with 50% showing clinical signs. None of the mice treated with finafloxacin (either for 7 or 14 days) lost any body weight p.c. (Figure 6A, significant days 7–9 p.c. *p <* 0.001, day 10, *p* = 0.011) or developed clinical signs of disease. Interestingly, those animals treated for 7 days gained weight faster (significant from day 14 p.c. compared to the carrier treated group (*p* < 0.05) and from day 26 p.c. compared to the animals treated with14 days of finafloxacin *p* < 0.05).

Treatment with 7 or 14 days of finafloxacin significantly reduced both the incidence of splenomegaly and increases in lung weight compared to organs from the control group (Figures 6B,C; lung day 8 p.c. *p* = 0.001; day 15 p.c. *p* = 0.003, spleen day 8 *p* = 0.014, day 15 *p* < 0.001, day 28, *p* = 0.023), although some splenomegaly was observed on day 28 p.c. irrespective of the length of the treatment regimen.

Treatment with finafloxacin also prevented the replication of *C. burnetii* within the lungs during the first 7 days (Figure 6D day 8 p.c. *p* < 0.05), however extended treatment prevented bacterial clearance. Between day 8 and day 15 p.c., the viable count in the lungs of the animals treated with the finafloxacin carrier reduced by over 100-fold (*p* < 0.001, mean day 8 p.c. 2 × 10^4^/g vs. day 15 p.c. 4.8 × 10^1^/g, *p* < 0.001) whereas the viable count in the lungs from animals treated with finafloxacin remained unchanged (mean day 8 p.c. 3.5 × 10^3^/g vs. day 15 p.c.1.1 × 10^3^/g) which resulted in a significantly higher level of bacterial colonization in the animals treated with finafloxacin (day 15 p.c., *p* < 0.01).

By day 35 p.c., 30% of the lungs from mice receiving the carrier were clear of viable bacteria whereas all of the lungs from mice treated with finafloxacin were still colonized, and the bacterial count was significantly higher (*p* < 0.05). Similarly, 7 days of treatment with finafloxacin prevented bacterial colonization of the spleen by day 8 p.c., with only 1 mouse colonized (Figure 6E). However, despite increasing the length of the finafloxacin treatment to 14 days, by day 15 p.c. 50% of the mice had viable bacteria in their spleen, although the bacterial load was lower than those receiving the diluent (*p* < 0.001). There was no difference in the bacterial load colonizing the spleens between any of the treatment groups at day 28 or day 35 p.c., (30% of mice treated with finafloxacin and 10% of the controls had viable bacteria in their spleens).

**FIGURE 7:**
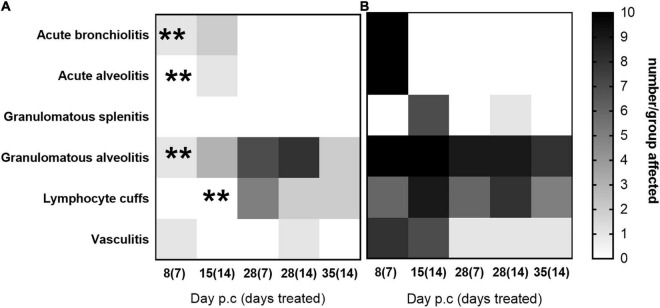
The occurrence of typical histopathological features associated with *C. burnetii* in lung and spleen tissues. At 24 h post-challenge mice were treated with **(A)** finafloxacin (30 mg/kg) or **(B)** the carrier, once a day orally for 7 or 14 days. Tissues were harvested on days 8, 15, 28, and 35 p.c. and scored for the presence/absence of 6 typical histopathological features associated with murine Q fever and displayed as a heat map (where white is no animal affected and black is all 10 affected). **p < 0.01.

Histopathological analyses was performed on sections of the lung and spleen samples and is shown in Figure 7. Tissues from animals receiving the carrier displayed all of the features associated with murine Q fever, and some resolution of this tissue pathology by day 28 p.c. The tissues from animals treated with finafloxacin had very few pathological lesions in the lung, and no damage observed in the spleen. Treatment for 14 days prevented the extent of the tissue damage observed on day 14 p.c. in the second study (detailed above) (Figure 5A), specifically the severity of granulomatous alveolitis (*p* < 0.05), lymphocyte cuffs and vasculitis (*p* < 0.001). Within the finafloxacin treated groups, the severities of all these histopathological features were determined to be minimal, there was no differences observed between the treatment regimens by day 28 p.c.

## Discussion

Doxycycline is the recommended antibiotic for the treatment of Q fever in humans (Anderson et al., 2013), however, it is poorly tolerated by some individuals and associated with a varied list of side effects (Järvinen et al., 1995; Maurin and Raoult, 1999; Saunders et al., 2015). Despite the majority of *C. burnetii* infections being self-limiting, there is a requirement for effective antibiotic treatment, as untreated, or poorly controlled disease, can lead to long term life-changing or life-threatening sequelae such as endocarditis or chronic fatigue syndrome (van Loenhout et al., 2012; Kampschreur et al., 2014). Recent advances in *C. burnetii* research, made possible by the development of an axenic growth media (Omsland et al., 2009), have led to the development of additional *in vitro* methods to enable the evaluation of antibiotic susceptibilities (Kersh, 2013; Clay et al., 2018; Smith et al., 2019). In addition, there are few *in vivo* efficacy studies that have been performed (Bewley, 2013; Eldin et al., 2017), and only recently have researchers started to use viable counts, rather than RT-PCR, as an alternative method to determine the level of bacterial colonization within tissues from animal models of Q fever (Hartley et al., 2019).

Here we report the evaluation of a novel fluoroquinolone, finafloxacin, in a murine model of inhalational Q fever. Treatment with finafloxacin was shown to reduce the severity of clinical signs of disease and weight loss associated with Q fever but did not reduce the level of bacterial colonization in tissues, when compared to treatment with doxycycline or ciprofloxacin. However, histopathological analysis performed on sections of the lungs and spleens suggests that treatment with finafloxacin reduced the presence of lesions typically associated with infection with *C. burnetii*. In addition, we report for the first time, the use of viable counts to evaluate antibiotic efficacy *in vivo*.

Doxycycline, a bacteriostatic tetracycline used to treat many respiratory infections, has reduced activity in acidic conditions (Smith et al., 2019). Ciprofloxacin, a bactericidal fluoroquinolone, is not a first line treatment for Q fever, but has been used to successfully treat Q fever endocarditis (Yebra et al., 1990). We have previously demonstrated that ciprofloxacin is not effective at treating murine Q fever (Norville et al., 2014). However, finafloxacin, although another fluoroquinolone, is unlike ciprofloxacin, in that it maintains antimicrobial activity at low pH (Higgins et al., 2010). As *C. burnetii* replicates entirely within an acidic vacuole *in vivo*, finafloxacin has the potential to be an effective treatment for Q fever.

The A/J mouse model of Q fever, is a non-lethal model that uses body weight loss as a key parameter to measure disease severity, combined with clinical signs of disease such as piloerection and the development of arched backs (Norville et al., 2014; Hartley et al., 2019). Mice receiving 7 days of treatment with finafloxacin, in our first study, were shown by day 14 p.c. to have lost less weight than those treated with 7 days of doxycycline or ciprofloxacin. Finafloxacin treated mice were also better protected against splenomegaly, another key disease feature observed in this mouse model of Q fever. However, by day 14 p.c. the mice treated with finafloxacin were colonized with a significantly higher bacterial load in the lungs and spleen compared to the finafloxacin carrier controls, determined by viable count but not by RT-PCR. GE is a measure of the total *C. burnetii* genomic content rather than viable organisms so the discrepancy between these 2 readings either relates to loss of viability or loss of cultivability of the bacteria in the carrier control group (Hartley et al., 2019). Doxycycline is normally prescribed for 14 days to treat Q fever in humans and shorter treatment regimens have been shown to delay the onset of disease, rather than reducing the severity (Benenson and Tigertt, 1956). The determination of the bacterial load by viable count rather than the historical method of RT-PCR (total DNA) appears to support this, and the pathogenesis study suggested that treatment with finafloxacin prevented bacterial replication and dissemination during the treatment period. Once treatment was stopped, the bacterial load increased in the lungs and spleens, but not to the pathological level observed in untreated mice. Mice have been shown to be tolerant of high levels bacterial colonization without displaying overt clinical signs of disease (Hartley et al., 2019), and this may, in part explain seroconversion in the 60% of asymptomatic human cases. It is interesting to note that on day 14 p. c., the mice treated with 7 days of finafloxacin (pathogenesis study) had enlarged organs, with viable bacteria exceeding 1 × 10^5^/g in the lung and 1 × 10^4^/g in the spleen. All animals had evidence of tissue damage but were not displaying any clinical signs of disease.

Extending treatment with finafloxacin from 7 to 14 days did not prevent the systemic dissemination of *C. burnetii* to the spleen, or result in a reduction in tissue damage by day 28 p.c. Although difficult to compare across studies due to a variation in infectious dose and therefore disease severity (determined by weight loss), extending the treatment period to 14 days did reduce the localized tissue damage which peaked at day 14 p.c. in the pathogenesis study. However, there was no reduction in the viable counts within the lung during the treatment period, compared to at least a 100-fold reduction in the viable count in the lungs from mice treated with the carrier. Currently there is no published literature detailing the assessment of the effect of finafloxacin on immune function, however fluoroquinolones have previously been shown to have an immunomodulatory effect (Dalhoff and Shalit, 2003). The high level of bacterial colonization may be a result of bacterial stasis achieved by the antibiotic, where the bacteria has been phagocytosed by macrophages, is contained within the lysosomes, effectively dormant, and is therefore not initiating an appropriate immune response (Voth and Heinzen, 2007). In addition, whilst the bacteria are contained within the macrophages they are able to be trafficked around the body allowing for the colonization of other organs. However, treatment with finafloxacin must result in some level of immune engagement, because once the treatment has ceased, and the bacteria can replicate, the level of disease that develops is well controlled. There is no evidence to suggest that finafloxacin has a bactericidal effect *in vivo*, as there is no reduction in the bacterial loads in the lung between days 4–8 and 7–14 days p.c. (during the treatment period). It is very likely that doxycycline causes the same effect. Studies have shown that the treatment of patients with doxycycline achieved a better result if delayed until the onset of symptoms, allowing the development of an adaptive immune response to be initiated (Benenson and Tigertt, 1956).

Evaluating the bacterial load by viable count raises another interesting question. In the final study (detailed above) by day 35 p.c. all of the mice treated with finafloxacin were colonized within the lung and 30% within the spleen, as determined by growth on axenic media. We do not know the relevance of this finding, it may be that day 35 p.c. is too early to demonstrate clearance. In human disease, IgG1 antibody levels start appearing around day 29, peaking around day 42 (Wielders et al., 2015) which presumably coincides with bacterial clearance. However, long term sequalae from Q fever have significant morbidity, and it seems very likely that poor treatment or uncontrolled disease are more likely to lead to a chronic outcome (Kampschreur et al., 2014). One long term assessment of aerosolized *C. burnetii* in Balb/c mice detected *C. burnetii* DNA in the lungs and spleens 8 weeks post-challenge (Melenotte et al., 2016). Although this does not necessarily indicate the presence of viable bacteria, it may be that *C. burnetii* can persist within the lung tissue for extended periods, a fact that would help explain the presence of viable bacteria reported by others in the bone marrow (Marmion et al., 2009) and the heart valves (Million et al., 2010).

Few studies have been carried out to investigate the development and resolution of *C. burnetii*-associated lesions identified in key tissues. Although this study did not determine whether any tissue damage observed was permanent (beyond 35 days p.c.), it has determined that in the A/J mouse, these lesions are most likely to be transient in nature. This was supported by the observation that organ weight to return to near normal. It also aligns with histopathological analysis from a range of tissues harvested from Balb/c mice infected with *C. burnetii*, which observed limited pathology on day 14, but not at 2 months p.c. (Melenotte et al., 2016).

We have demonstrated that finafloxacin is superior to doxycycline for the treatment of Q fever in a murine model, resulting in the reduction of clinical signs of disease and tissue damage. It also appears to be better tolerated than doxycycline by the mice. This is important as chronic Q fever in humans is treated for extended periods (often years) with doxycycline. Unfortunately, there is currently no *in vivo* model to determine antibiotic efficacy for chronic Q fever (Bewley, 2013; Dragan and Voth, 2020), but finafloxacin would appear to be an excellent candidate.

## Data Availability Statement

The original contributions presented in the study are included in the article/supplementary material, further inquiries can be directed to the corresponding author/s.

## Ethics Statement

The animal study was reviewed and approved by the DSTL animal ethics committee, following the United Kingdom Animal (Scientific Procedures) Act (1986) and the Codes of Practice for the Housing and Care of Animals used in Scientific Procedures, 1989.

## Author Contributions

All authors listed have made a substantial, direct, and intellectual contribution to the work, and approved it for publication.

## Author Disclaimer

This material is licensed under the terms of the Open Government Licence except where otherwise stated. To view this licence, visit http://www.nationalarchives.gov.uk/doc/open-government-licence/version/3 or write to the Information Policy Team, The National Archives, Kew, London TW9 4DU, or email: psi@nationalarchives.gov.uk.

## Conflict of Interest

The authors declare that the research was conducted in the absence of any commercial or financial relationships that could be construed as a potential conflict of interest.

## Publisher’s Note

All claims expressed in this article are solely those of the authors and do not necessarily represent those of their affiliated organizations, or those of the publisher, the editors and the reviewers. Any product that may be evaluated in this article, or claim that may be made by its manufacturer, is not guaranteed or endorsed by the publisher.
